# Prevalence of *Salmonella* spp. and *Staphylococcus aureus* in Chicken Meat and Pork from Cambodian Markets

**DOI:** 10.3390/pathogens10050556

**Published:** 2021-05-04

**Authors:** Chea Rortana, Hung Nguyen-Viet, Sothyra Tum, Fred Unger, Sofia Boqvist, Sinh Dang-Xuan, Sok Koam, Delia Grace, Kristina Osbjer, Theng Heng, Seng Sarim, Or Phirum, Roeurn Sophia, Johanna F. Lindahl

**Affiliations:** 1 National of Animal Health and Production Research Institute, General Directorate of Animal Health and Production, Phnom Penh 12100, Cambodia; sothyratum@gmail.com (S.T.); sok.koam@yahoo.com (S.K.); theng.heng11@gmail.com (T.H.); sengsarim87@gmail.com (S.S.); phirumor@gmail.com (O.P.); roeurnsophai24@gmail.com (R.S.); 2 International Livestock Research Institute, Nairobi 00100, Kenya; h.nguyen@cgiar.org (H.N.-V.); F.Unger@cgiar.org (F.U.); S.Dang@cgiar.org (S.D.-X.); d.randolph@cgiar.org (D.G.); 3 Department of Biomedical Science and Veterinary Public Health, Swedish University of Agricultural Sciences, 75007 Uppsala, Sweden; Sofia.Boqvist@slu.se; 4 Center for Public Health and Ecosystem Research, Hanoi University of Public Health, Hanoi 100000, Vietnam; 5 Natural Research Institute, University of Greenwich, Kent ME4 4TB, UK; 6 Emergency Centre for Transboundary Animal Diseases, Food and Agriculture Organization of the United Nations, Phnom Penh 12100, Cambodia; Kristina.Osbjer@slu.se; 7 Department of Clinical Sciences, Swedish University of Agricultural Sciences, 75007 Uppsala, Sweden; 8 Department of Medical Biochemistry and Microbiology, Uppsala University, 75123 Uppsala, Sweden

**Keywords:** animal-source food, Cambodian traditional market, food safety, livestock product, *Salmonella* species, *S. aureus*, wet market

## Abstract

*Salmonella* spp. and *Staphylococcus aureus* are two of the most common foodborne bacteria in animal-source foods (ASF) that cause illness worldwide. This study aimed to determine the prevalence of *Salmonella* spp. and *S. aureus* in chicken meat and pork in markets in Cambodia. Sampling was done in 52 traditional markets and 6 supermarkets in 25 provinces of Cambodia between October 2018 and August 2019. In total, 532 samples were obtained: chicken meat and pork (n = 408, 204 of each), chicken and pork cutting board swabs (n = 124, 62 of each). All samples were analyzed for the presence of *Salmonella* spp. and *S. aureus*; colony-forming units per gram (CFU/g) of coagulase-positive *Staphylococci* (CPS) were counted, and a subset of samples was also analyzed for the most probable number (MPN, n = 136) of *Salmonella*. The overall prevalence of *Salmonella* spp. and *S. aureus* were 42.1% (224/532) and 29.1% (155/532), respectively, with 14.7% (78/532) of samples containing both bacteria. The prevalence of *Salmonella* spp. in chicken meat was 42.6%, on chicken cutting board it was 41.9%, on pork it was 45.1%, and the pork cutting board 30.6%. Chicken meat had a significantly (*p-*value < 0.05) higher prevalence of *S. aureus*, 38.2%, compared to the chicken cutting board, 17.7%, pork 28.9%, and pork cutting board 11.3%. Mean MPN-*Salmonella* was 10.6 MPN/g in chicken and 11.1 MPN/g in pork samples. Average Log CFU/g of CPS in chicken and pork samples were 2.6 and 2.5, respectively. The results indicate that chicken meat and pork in Cambodia were highly contaminated with *Salmonella* spp. and *S. aureus*, posing risks to consumers’ health. Urgent interventions are necessary to improve hygiene for safer meat in Cambodian markets.

## 1. Introduction

Foodborne diseases (FBD) are the illness conditions caused by the ingestion of food containing biological, chemical, or physical hazards. Biological hazards such as bacteria, virus, parasites are responsible for most illnesses. FBD constitute a significant threat to health and impediments to social and economic development worldwide, especially in low- and middle-income countries (LMIC) [1,2,3]. Foodborne disease is one of the leading causes of human mortality and morbidity, comparable to major infectious diseases such as malaria, HIV/AIDS and tuberculosis [4]. Based on a comprehensive review of 31 common microbes causing FBD worldwide, the World Health Organization (WHO) estimated the health burden of 26 priority hazards at 33 million Disability Adjusted Life Years (DALYs); with an exceptionally high (40%) burden in children under five years of age [5]. Older people and people with chronic disease and children under five years of age are the most susceptible to FBD [6,7]. The group most at risk of FBD comprises those living in LMIC, high population density, limited knowledge, and lack of good hygiene practices for fresh meat handling [1,6,7]. A related study, also by the WHO, estimated an additional burden of 9 million DALYs associated with four heavy metals in food [8]. A recent study estimated a loss of more than US$110 billion in productivity and medical expenses each year from unsafe food in LMIC [9]. However, few LMICs monitor the presence of FBD, and thus, data on the burden are limited, while more data are available in high-income countries [1,3,9,10].

Animal-source foods (ASF) provide essential nutrients for humans in palatable and digestible forms; however, they also act as a transmission route for common foodborne pathogens and toxins produced by microbes [11,12]. Bacteria are the leading causes of foodborne illness, particularly diarrheal disease [13,14]. Foodborne bacteria can infect humans by consumption of raw and under-cooked products but may also cross-contaminate ready-to-eat food [15].

*Salmonella* spp. and *Staphylococcus aureus* are two of the most common foodborne bacteria in ASF. *Salmonella* is a genus of Gram-negative, rod-shaped bacteria with a facultative metabolism. There are two common ASF-associated species of *Salmonella*, including *S. enterica* and *S. bongori,* with almost all *S. enterica* associated with human salmonellosis. *Salmonella* spp. cause a variety of diseases in humans and animals [16]. Non-typhoidal *Salmonella* are among the most important causes of diarrheal in humans, contributing to an estimated 230,000 deaths annually [5,17]. *Salmonella* spp. can contaminate fresh meat during slaughter or processing, handling, and during selling at the markets [18,19]. In livestock, such as pigs and chickens, *Salmonella* spp. colonization can be subclinical and difficult to detect by animal inspectors before slaughter but may contaminate carcasses and infect humans via consumption [18,20].

*Staphylococcus aureus* is a Gram-positive bacterium regarded as human commensal. It is also an opportunistic pathogen that can cause a broad spectrum of infections, from superficial skin infections to severe, and potentially fatal, invasive disease [21,22]. *S. aureus* and some of the coagulase-positive staphylococci (CPS) species are human pathogens, causing a wide range of clinical signs, including foodborne illness, by its wide range of enterotoxins production [23,24,25]. Most of the Staphylococcal enterotoxins are mostly heat resistant and can cause human diseases via consumption of contaminated food even if properly cooked [12,23]. *S.* *aureus* is frequently isolated from meat and ready-to-eat foods [22,24,26,27]. The prevalence of *S. aureus* in meat products needs to be monitored and controlled in LMICs, including Cambodia [1,19,22,28].

Commonly for daily consumption, most Cambodians purchase fresh food, especially fresh meat, from traditional markets, sometimes referred to as wet markets [29]. In these markets, local people buy and sell products, especially ASF, such as fresh pork, poultry, fish, fresh vegetables, and basic household commodities [30,31]. Generally, traditional markets in Cambodia are similar to those in nearby countries such as Lao PDR and Vietnam, where ASF safety is still below satisfactory [19,32,33]. Earlier studies found that hygiene practices in slaughterhouses and among meat retailers in Cambodia were not well, and the methods of handling and slaughtering followed traditional practices that were not always hygienic. For example, the slaughtering process was mainly done on the floor, and the personnel hygiene of workers was not well managed [18,20]. In addition, the basic slaughterhouse facilities and unhygienic handling and transportation of meat could contribute to contamination by microbes through the food chain to both the formal and informal retail market. Several risk factors are contributing to bacterial contamination and growth in carcasses/meat, including poor infrastructure, lack of cleaning and disinfection, unhygienic handling of contaminated materials, and lack of temperature control [30].

Retail meats sold in supermarkets can be safer than meat sold in traditional markets since supermarkets often have access to clean water, cooling systems and appropriate processing, but in Cambodia, supermarkets are uncommon. According to the Cambodian Annual Report of Animal Health and Production in 2019, there were 480 traditional markets that serve and sell fresh meat for most people countrywide, and only a few supermarkets and minimarts selling different types of meat [28]. The objective of this study was to determine the prevalence of *Salmonella* spp. and *S. aureus* in chicken meat and pork and cutting boards for chicken and pork in Cambodian traditional markets and supermarkets, the information needed for food safety management.

## 2. Results

### 2.1. Prevalence of Salmonella *spp.* and Staphylococcus aureus in Food Samples Collected at Cambodian Markets

#### 2.1.1. Overall Prevalence

The study comprised 532 samples from 52 traditional markets and 6 supermarkets in 25 provinces/municipalities of Cambodia (Table 1 and Table 2). In total, 42.1% (224/532) of the samples were positive for *Salmonella* spp. and 29.1% (155/532) were positive for *S. aureus* (Table 2). Among these, 14.7% (78/532) of the samples were positive for both *Salmonella* spp. and *S. aureus*. The prevalence of both bacteria in meat samples (chicken and pork) was significantly higher than that on cutting boards used for chicken and pork (*p-*value < 0.001). The bacterial contamination of all sample types (chicken meat and pork) from supermarkets was lower than that from traditional markets (*p-*value = 0.002). There was a notable variation in microbial contamination between provinces/municipalities (Table 1).

#### 2.1.2. Traditional Markets

The prevalence of both *Salmonella* spp. and *S. aureus* across all samples was 16.3% (68/416), while in chicken meat it was 20.5% (32/156), on chicken cutting boards 9.6% (5/52), in pork 19.2% (30/156) and on pork cutting boards 1.9% (1/52) (Table 2). The prevalence of *Salmonella* spp. in chicken meat was 40.4% (63/156), on chicken cutting boards 42.3% (22/52), in pork 45.7% (70/156), and on pork cutting boards 11.3% (14/52). In comparison between the two species, the prevalence of *Salmonella* spp. in chicken and pork samples (including cutting boards of both sample types) was not significantly different (*p-*value = 0.15). The prevalence of *S. aureus* in chicken meat was 46.2% (72/156), on chicken cutting boards 21.2% (11/52), in pork 34.6% (54/156), and on pork cutting boards 13.5% (7/52). The prevalence of *S. aureus* was significantly higher in chicken samples than in pork samples (*p-*value < 0.001).

#### 2.1.3. Supermarkets

Among the 36 samples from six supermarkets (Table 2), the prevalence of *Salmonella* spp. was 16.7% (3/18) in chicken and 38.9% (7/18) in pork. *Staphylococcus aureus* was not found in chicken and only in 5.6% (1/18) of pork samples. Only one pork sample was positive for both *Salmonella* spp. and *S. aureus* (1/18, 5.6%).

#### 2.1.4. Variation in Prevalence within One Year

**Figure 1:**
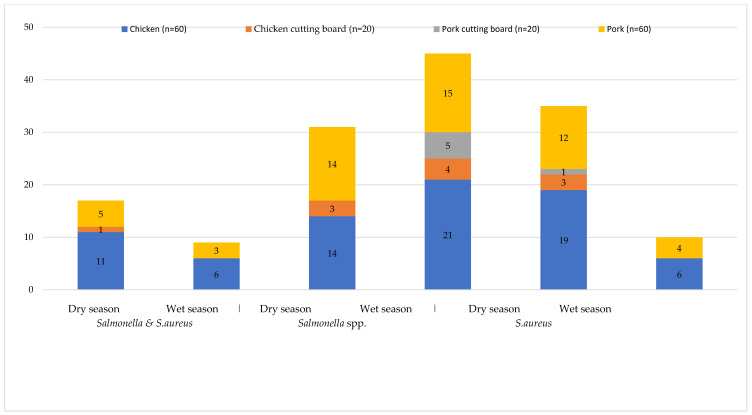
Number of positive samples for *Salmonella* spp. and *Staphylococcus aureus* in the wet and dry seasons in Cambodia. Number of samples (included chicken, chicken cutting board, pork cutting board and pork) per season were 80. The dry and wet seasons in Cambodia are from November to April and May to October, respectively.

During the repeated sampling in the dry season, the prevalence of co-contamination with *Salmonella* and *S. aureus* was 20.0% (6/30) in chicken and in pork 10.0% (3/30), no cutting boards being positive for co-contamination. The prevalence of *Salmonella* spp. in chicken meat was 70.0% (21/30), on chicken cutting boards 40.0% (4/10), in pork 50.0% (15/30), and the pork cutting boards 50.0% (5/10) (Figure 1). *S. aureus* was found only in chicken meat and pork at a frequency of 20.0% (6/30) and 13.3% (4/30), respectively (Figure 1).

In four provinces, samples were collected in both dry and wet seasons (Figure 1). In the total number of samples, the prevalence of co-contamination with *Salmonella* spp. and *S. aureus* in the dry season was 21.3% (17/80) and 11.3% (9/80) in the wet season. The prevalence of *Salmonella* spp. in all sample categories in the wet season was 56.3% (45/80), which was significantly higher than in the dry season 38.8% (31/80, *p-*value = 0.01). The prevalence of *S. aureus* in the dry season was 43.8% (35/80), which was significantly higher than in the wet season at 12.5% (10/80, *p-*value < 0.001, Table 2).

#### 2.1.5. Factors Associated with Prevalence of *Salmonella* spp. and *Staphylococcus aureus* Contamination

The multivariable analyses showed significantly lower prevalence in the supermarket when compared to traditional markets regarding the prevalence of both *Salmonella* spp. and *S. aureus* (*p-*value = 0.034) and with only *S. aureus* (*p-*value = 0.002). The prevalence of *Salmonella* was not significantly different between these two market types (*p-*value = 0.09). The prevalence of *S. aureus* was significantly higher (*p-*value < 0.001) in meat samples than in cutting boards. There was also a tendency for higher *Salmonella* spp. prevalence in meat samples (*p-*value *=* 0.07). The prevalence of *Salmonella* spp. increased during the wet season, while the prevalence of *S. aureus* was the opposite (Table 3).

**Figure 2:**
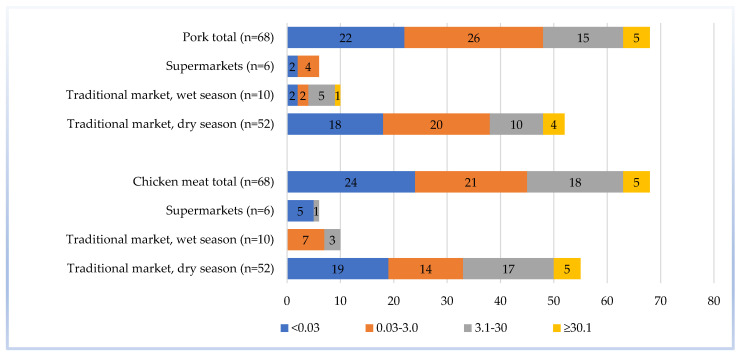
Frequency of *Salmonella* spp. most probable number (MPN/g) ranges in meat samples (n = 136) collected from Cambodian markets.

Of the 136 selected samples, the *Salmonella* MPN/g indexes were divided into four groups: <0.03, 0.03–3.0, 3.1–30, and ≥30.1. Most of the pork and chicken samples ranged from <0.03 to 0.03–3.0 MPN/g. Meat samples from traditional markets had the highest *Salmonella* MPN/g range (≥30.1), which were mainly found in the dry season. While in the wet season, the highest *Salmonella* MPN/g range was only found in pork samples. Both pork and chicken samples collected from supermarkets did not exceed 30.0 MPN/g (Figure 2).

### 2.2. Coagulase-Positive Staphylococci

**Figure 3:**
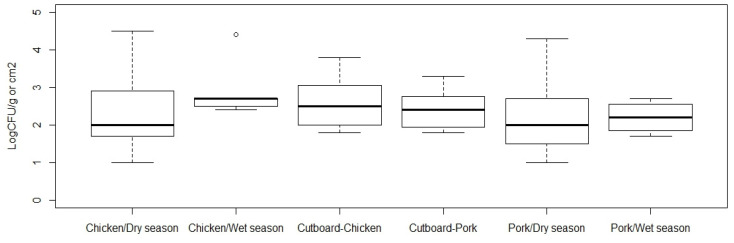
Contamination of coagulase-positive staphylococci (Log CFU/g or cm^2^) in samples collected from Cambodian traditional markets in dry and wet seasons. Cutting board samples in chicken and pork shops were only collected in the dry season.

All samples in the traditional market were tested and quantified (CFU/g) for CPS. An average Log CFU/g of CPS from chicken meat and pork samples was higher in wet season compared to dry season, 2.3 (SD 1.0) versus and 2.8 (SD 0.7) in chicken, and 2.1 (SD 0.9) versus 2.2 (SD 0.4) in pork. An average Log CFU/g of CPS contaminated on cutting board was similar in chicken and pork shops (Figure 3). Results from linear regression showed that the CPS contamination in meat in supermarkets was lower than in traditional markets (*p-*value < 0.001; Table 4). Regarding meat types, the load of CPS in chicken was significantly higher than in pork (*p-*value = 0.017), whereas the load of CPS in meat was significantly higher than in cutting board (*p-*value < 0.001, Table 4).

## 3. Discussion

The main objective of this research was to assess the prevalence of two important human pathogens in meat sold in Cambodia, mostly in traditional markets, to understand the risks for consumers and inform interventions for improving hygiene practices for safer ASF retail. This is the first nationwide survey in traditional markets in all 25 provinces/municipalities and in supermarkets of Cambodia. Our study found a high prevalence of both *Salmonella* spp. and *S. aureus* in all market types.

The overall prevalence of *Salmonella* spp. of this study was 42.1%, with similar contamination rates in both chicken and pork. *Salmonella* spp. is one of the most common foodborne pathogens in fresh meat in Southeast Asia [2,14]. The *Salmonella* spp. prevalence found in this study in Cambodia is similar to that in Vietnam, where some recent studies reported a *Salmonella* spp. prevalence of 45.9% out of 900 chicken samples [34] and 44.7% out of 217 pork samples [33]. An earlier study from the border of Cambodia–Thailand reported a 23% prevalence of *Salmonella* spp. in chicken meat from 145 samples [35]. Another study reported a much higher prevalence of *Salmonella* spp. of 88.2% from 152 poultry carcasses, randomly selected from 10 markets in retail outlets of Phnom Penh between March 2006 and February 2007 [36]. However, our study found a large variation among 25 provinces/municipalities, with some having less than 20% of chicken samples contaminated, and others more than 75%. This result indicates that the prevalence may vary considerably among provinces. We also found seasonal variation in prevalence. Another study conducted in Bangkok, Thailand, found that the prevalence levels of *Salmonella* spp. in chicken collected from open markets and supermarkets were 48% (n = 61) and 57% (n = 75), respectively [31]. Although our study indicated that supermarkets had a lower prevalence of *Salmonella* spp. contamination than in traditional markets, the supermarket prevalence was still at an unacceptable level. Moreover, as the number of samples from supermarkets was small, expanding future surveys on the foodborne pathogen in chicken meat and pork in the supermarket is recommended.

The present study showed that the MPNs for *Salmonella* spp. in fresh chicken meat and pork mainly ranged from <0.03 to 30 MPN/g. An earlier study in Phnom Penh, Cambodia, found a varied concentration of *Salmonella* from 10 to 10^4^ CFU/g [36]. Comparable to our results, a study in China on the *Salmonella* quantity in chicken meat showed that more than half of *Salmonella* samples had higher than 0.7 MPN/g [37]. In Vietnam, *Salmonella* concentration in cut pork from traditional markets was mainly lower than 3.0-30 MPN/g, which is at similar contamination ranges compared to our findings in pork [33]. There was a similar concentration of *Salmonella* spp. in Cambodia and Vietnam, which might be due to a similar slaughterhouse environment and transportation. The fact is that bacteria are more likely to grow well during the selling period without temperature control [38].

*S. aureus* was found among both meat samples and cutting boards of both meat types, which shows that the pathogen is present in fresh meat and its environment in Cambodian markets. CPS are among the major foodborne pathogens that produce enterotoxins which could persist even when products are well cooked and are the etiological agents of staphylococcal food poisoning [24]. There was a slight difference in the prevalence of bacteria found in chicken and pork in traditional markets in 25 provinces/municipalities of Cambodia, which could be due to different hygiene practices. The contamination of *S. aureus* was more common in the dry season, which could be explained by the fact that the wet markets have high moisture and temperature, stimulating the growth of this pathogen in meat. In 2014, *S. aureus* was reported as the cause of gastroenteritis from the FBD outbreak in rural Cambodia. Those cases were due to poor personal hygiene and handwashing, and cross-contamination from other raw animal products [27]. Another study presented a high *S. aureus* contamination rate in Vietnamese ready-to-eat food, ranging from 12.5% to 35.4%, and the contamination in milk was the highest [26]. Another study found that about 40% (18/45) of these isolates having classical *S. aureus* and staphylococcal enterotoxins pose threats to human health [39]. These results indicate the importance of CPS for human health, not only in Cambodia but the whole region. A previous study found an acceptable number of *S. aureus* in beef products in supermarkets in Cambodia [40] but did not test pork or chicken. However, in general, this pathogen has only been little studied in food in Southeast Asia [14].

This study indicates that *Salmonella* spp. contamination was more common during the wet season when increased moisture and water on handling equipment could facilitate *Salmonella* spp. contamination of meat. In the wet season, Cambodia has a high humid condition, which could increase the survival of *Salmonella* in the market, where there is a tradition of selling meat at the shop without temperature control. A study from Denmark in the past decades also found that seasons with higher rainfall can support the survival of *Salmonella* spp. and increase contamination of meat carcass during slaughtering, transportation, and at retail [41].

Previously, FBDs were known collectively as “diarrheal diseases” rather than caused by specific foodborne pathogens. Recently, however, the Foodborne Disease Burden Epidemiology Reference Group (FERG) of WHO reviewed FBD as a distinct category based on secondary data, but the exact source of microbial contamination in food remains limited available in many LMIC [5]. The FERG found that around half the burden of FBD was due to diarrhea, the rest being caused by less common but more severe illnesses such as epilepsy, congenital disabilities, and arthritis. The current Cambodian food safety standard for animal-source food such as chicken and pork requires less than 50,000 CFU/g of total bacteria count, *Salmonella* spp. free in 25 g of meat, and <100 CFU/g of CPS [42]. However, due to lack of resources, the current inspection practices are based on hygienic indicators, TBC and *Salmonella* spp., but not limited to other pathogens such as *S. aureus*. The present study found that 42.1% of meat contained *Salmonella* and 29.1% contained *S. aureus*, showing meat contamination higher than the current standards. The results suggest the need of improving hygienic practice at markets, as well as food safety awareness of meat sellers, to reduce the risk of FBD. Successful interventions in retailer markets have been reported in Vietnam, Malaysia, and African countries [32,43,44,45]. An example from Vietnam shows that food safety research and evidence of bacterial contamination can attract much attention from media and scientists and inform the government, leading them to adopt a risk-based approach to manage food safety [32,46]. In addition, the study in Malaysia suggests the need for enforcement of legislation and regulations and improvement of public–private partnership in the food system [45]. According to studies in African countries, a powerful method for improving food safety in the informal market was applying risk-based approaches and intense collaboration of local and international institutions [1,43,44]. Our study provides local data on microbial contamination in chicken and pork in both traditional and modern markets, which will help inform consumers about the public health risks. The result will also be an important message to food safety policy makers to improve risk management and risk communication.

Finally, this study focused mainly on sampling in traditional markets where more than 90% of the food was traded for Cambodia and collected only a few samples from supermarkets in the two largest cities. Recent discussion at the global level on market types showed that ASF from supermarkets was not necessarily safer than traditional markets [32]. However, in this study, pork and chicken from supermarkets had lower levels of samples contaminated with *S. aureus* or *Salmonella* spp. and even both pathogens. Although the number of samples from supermarkets is small (36 samples versus 416 from traditional markets), this shows a promising trend in food safety correlated with the formalization of markets in demand for food in Cambodia. Interestingly, supermarkets were relatively better performing with *S. aureus* than *Salmonella* spp.; the former is often associated with poor handling and hygiene, while the latter may be more related to contamination at production. In contrast, the low prevalence of *S. aureus* in the supermarket may be associated with appropriate temperature control, a clean water system, and handling practices.

In conclusion, this study found a high prevalence of both *Salmonella* spp. and *S. aureus* in chicken meat and pork samples, which could cause serious FBD in humans. Vulnerable people who consume fresh chicken meat and pork purchased from the traditional market might be at risk of contracting FBD. These pathogens may contribute to common foodborne illness in Cambodia, and interventions to improve hygienic practices in markets are strongly recommended. Policies engagement of local government is vital for the success of intervention and reduction of FBD.

## 4. Materials and Methods

### 4.1. Study Design and Sampling Frame

This cross-sectional study was carried out between October 2018 and August 2019. The first part of sampling was conducted during the dry season, October 2018 to May 2019, at Cambodian traditional markets selling meat products. This included two medium (i.e., having 15 to 50 meat sellers) or large (i.e., having more than 50 meat sellers) traditional markets in each of the 25 provinces/municipalities of Cambodia, except for the two cities with the largest population (Phnom Penh and Siem Reap), where three markets were included. The two traditional markets were the largest markets in each province identified by provincial veterinary authorities. In total, 52 traditional markets were included in the study. At each market, three pork and three chicken meat sellers were selected for sampling using systematic random sampling by the shop’s location in the meat selling area at the market, beginning from the main entrance gate, middle, and around the end. Among the three shops where chicken or pork was sampled, only one shop was selected for sampling cutting board swabs. A total of 416 samples were collected in this first part of sampling, representing the dry season.

The second part was a repeated sampling approximately five months after the first part of sampling and was conducted during the wet season from July to August 2019. The sampling was done only in four provinces/municipalities: Battambang, Phnom Penh, Siem Reap, and Preah Sihanouk. This repeated sampling targeted the same number of samples as in the first part of sampling (in the dry season) and generated a total of 80 samples.

The third part of sampling was conducted in supermarkets in October 2018, including four supermarkets in Phnom Penh and two in Siem Reap. Three chicken and three pork samples were purchased from each supermarket. A total of 36 meat samples, but no cutting board samples, were collected. The detailed sampling frame is shown in Table 5.

### 4.2. Sample Collection

This study aimed to assess the consumer exposure risk by obtaining samples following the ways customers would buy. Chicken meat and pork were purchased from the selected shops with approximately 300–400 g of each. The vendors used their knife and cutting board to cut the meat and their scale for weighing before placing it into the sterilized sampling bag. In addition, for one pork vendor and one poultry vendor per market, 100 cm^2^ of cutting board surface (the most common site used to cut meat) were swabbed. Swab samples were collected using a pre-moisturized sterilized cotton bandage compress, a 10 × 10 cm stainless frame, and a sterilized pincer and were placed in a sterilized plastic zip-lock bag containing 10 mL normal saline. The study excluded the co-contamination of bacteria from hand retailers and all their equipment attached with meat at the shop. The samples were stored in cooling boxes and transported to the laboratory within 24 h by field staff. All the tests were done at the bacteriology laboratory at the National Animal Health and Production Research Institute, General Directorate of Animal Health and Production, Phnom Penh, Cambodia.

### 4.3. Bacteriological Analysis

#### 4.3.1. *Salmonella* spp. Isolation

*Salmonella* spp. isolation followed the ISO procedure ISO-6579:2002/amended:1:2017 [47,48]. Each of the meat (chicken meat and pork) samples was sliced into small pieces aseptically, and 25 g were diluted in 225 buffered peptone water (BPW; Merck, Darmstadt, Germany) and homogenized using stomacher (Seward Limited, West Sussex, UK) for 2 min. For cutting board swab samples, which already contained 10 mL of liquid samples, 90 mL BPW were added and then homogenized manually. The suspensions of the meat sample and cutting board swabs were incubated for 16–20 h at 37 °C for pre-enrichment. Selective enrichment step was done by pipetting 1 mL aliquot in 9 mL Muller Kauffmann Tetrathionate (MKTT; Merck, Darmstadt, Germany) incubated for 16–20 h at 37 °C, and 0.1 mL aliquot in 10 mL Rappaport-Vassiliadis Soya (RVS; Merck, Darmstadt, Germany) incubated for 16–20 h at 41.5 °C. The selective plating was performed by one loop full (approx. 10 μL) of each MKTT and RVS onto Xylose-Lysine Deoxycholate Agar (XLD; Hi-Media, Mumbai, India) and MacConkey agar (Merck, Darmstadt, Germany) as the second plating-out medium. Five presumptive *Salmonella* colonies, with darker pink center or yellow with or without blackening, were subcultured on nutrition agar at 37 °C overnight for biochemical tests. Biochemically, *Salmonella* spp. were confirmed using lactose, indole production, lysine decarboxylase, H_2_S production, and urease.

#### 4.3.2. Most Probable Number of *Salmonella*

One-third of total meat samples (n = 124), including pork (n = 62) and chicken meat (n = 62), were selected for quantification of *Salmonella* spp. using a traditional 3-tube MPN method described previously [49]. In brief, each of the 25 g samples was suspended in 225 mL of PBW. From each dilution, 1 mL was added serially to each of 3 × 9 mL of BPW, thus creating a set of three MPN tubes with the dilutions of 10^−1^, 10^−2^, and 10^−3^. Pre-enrichment was followed by incubated (37 °C for 24–48 h) and transferred (one drop) to a corresponding 24-well plate containing 2.5 mL Modified Semi-Solid Rappaport-Vassiliadis (MSRV; Merck, Germany) and then incubated (41.5 °C for 24 h). *Salmonella* was confirmed by subculturing onto XLD agar (37 °C for 24 h), and biochemical tests were followed as mentioned above. MPN index was recorded according to De Man [50] and the bacteriological analytical manual [51].

#### 4.3.3. Isolation of Coagulase-Positive Staphylococci and *Staphylococcus aureus*

All samples were tested for the presence/absence and enumeration of coagulase-positive staphylococci (CPS) following the ISO 6888-1:1999 (includes amendment A1: 2003) using Baird-Parker (BP; Oxoid, Milan, Italy) agar medium [52,53]. In brief, each of the 25 g of pork or chicken meat samples was weighed, cut, and homogenized in 225 mL BPW. Each of the swab samples, approximately 10 mL, was added to 90 mL of BPW to produce the 10^−1^ dilution. Then, the diluted samples were aliquoted to a new 15 mL tube to produce the series of 10-fold dilution from 10^−1^ to 10^−3^. Then, 0.1 mL aliquoted suspension was transferred and streaked on to two BP agar plates. The plates were then incubated at 37 °C in aerobic atmosphere. After 48 h, plates were examined to find the typical presumptive colonies with opaque and atypical without opaque. Both typical and atypical colonies were counted and calculated for the number of presumptive CPS. About 5 typical colonies were selected for the coagulase test using rabbit serum plasma (BD, USA). An equation [*Ne = Suma/(V(n*1* + *0.1*n*2*)d*] from ISO-6888-1-1999 for calculation of the number N of identified CPS present in the test proportion. After confirmation as coagulase-positive, the number of CPS were calculated according to the instruction in 10.1.1 of ISO 6888-1:1999. Colonies of CPS were streaked on to nutrition agar plates for growth at 37 °C for 24 h for further *S. aureus* confirmation using gram stain (Merck, Germany), oxidase test (Merck, Darmstadt, Germany), catalase test, and latex agglutination (Biomerieux SA, Craponne, France) [54].

### 4.4. Data Management and Analysis

All data were entered in Microsoft Excel. The relation of prevalence of the different sample types and bacteria were calculated using Pearson Chi-square. Multi-level logistic regression was the method for comparison between prevalence of bacteria with market type, seasons, sample types and species. The prevalence of *Salmonella* spp. in chicken meat, chicken cutting board, pork meat and pork cutting board by provinces/municipalities was analyzed using logistic regression. The number of colony-forming units for CPS were converted to Log CFU/g with the value zero substituted with 1, to generate a more normal distribution, and compared between CFU/g of CPS using linear regression. All statistical analyses were performed in EpiInfo^TM^, an open-source domain of software tools (CDC, USA) and RStudio (R core team). A p-value of 0.05 was used for statistical significance, with no compensation for multiple comparisons.

### 4.5. Ethical Consideration

Ethnical approval for meat specimen collection was received from the General Directorate of Animal Heath and Production, dated 12 October 2018. Ethical approval for retailer interviews was received from the National Ethical Committee of Cambodia, coded 300NECHR, dated 26 December 2017. Compliance for testing of sample and biosafety was approved by International Livestock Research Institute in letter ref: ILRI, RC-010-18/IBC/010/CR, dated 5th July 2018.

## 5. Conclusions

The study found a high prevalence of *Salmonella* spp. and *S. aureus* in chicken and pork samples, which can cause severe foodborne diseases in humans. These pathogens may contribute to common foodborne illness in Cambodia. Interventions to improve hygienic standards in Cambodian markets are strongly recommended in the traditional markets in provinces/municipalities with higher contamination levels. Further studies on how *Salmonell*a spp. and/or *S. aureus* could cross-contaminate to ready-to-eat food or any typical food in Cambodian households are suggested.

## Figures and Tables

**Table 1:** Prevalence of *Salmonella* spp. and *S. aureus* in chicken, chicken cutting boards, pork and pork cutting boards in Cambodian traditional markets by province.

Provinces/Municipalities	Markets ^1^	Total Sample Collected ^2^	Total Positive Samples	Number of *Salmonella* Positive Samples (%)					Number of *S. aureus* Positive Samples (%)				
				Chicken	Cutting Board Chicken	Cutting Board Pork	Pork	Average ^4^ MPN/g	Total Positive Samples	Chicken	Cutting Board Chicken	Cutting Board Pork	Pork
Phnom Penh	3 (2 times)	48	13 (27.1)	8 (44.4)	1 (16.7)	0 (0%)	4 (22.2)	16.1	12 (25.0)	5(27.8)	1 (16.6)	1 (16.6)	5 (27.8)
Siem Reap	3 (2 times)	48	31 (64.6)	14 (77.8)	3 (50.0)	1 (16.7)	13 (72.2)	2.6	12 (25.0)	8 (44.4)	0	0	4 (22.2)
Battambang	2 (2 times)	32	14 (43.8)	4 (33.3)	2 (50.0)	2 (50.0)	6 (50.0)	5.9	10 (31.3)	5 (41.7)	2 (50.0)	0	3 (25.0)
Preah Sihanouk	2 (2 times)	32	18 (56.3)	9 (75.0)	1 (25.0)	2 (50.0)	6 (50.0)	25.4	11 (34.4)	7 (58.3)	0	0	4 (33.3)
Takeo	2	16	8 (50.0)	3 (50.0)	1 (50.0)	1 (50.0)	3 (50.0)	15.7	5 (31.3)	2 (33.3)	1 (50.0)	0	2 (33.3)
Kampong Cham	2	16	5 (31.3)	1 (16.7)	1 (50.0)	0	3 (50.0)	15.0	10 (62.5)	5 (83.3)	1 (50.0)	1 (50.0)	3 (50.0)
Tboung Khmum	2	16	7 (43.8)	2 (33.3)	1 (50.0)	1 (50.0)	3 (50.0)	8.3	6 (37.5)	3 (50.0)	0	0	3 (50.0)
Kep	2	16	10 (62.5)	3 (50.0)	1 (50.0)	0	6 (100)	58.6	4 (25.0)	1 (16.7)	0	0	3 (50.0)
Kampot	2	16	10 (62.5)	3 (50.0)	1 (50.0)	1 (50.0)	5 (83.3)	55.2	5 (31.3)	4 (66.7)	0	0	1 (16.7)
Kampong Speu	2	16	6 (37.5)	3 (50.0)	0	0	3 (50.0)	3.5	11 (68.8)	6 (100)	0	0	5 (83.3)
Kandal	2	16	6 (37.5)	1 (16.7)	1 (50.0)	2 (100)	2 (33.3)	107.5	3 (18.8)	3 (50.0)	0	0	0
Kampong Chhnang	2	16	9 (56.3)	4 (66.7)	2 (100)	0	3 (50.0)	51.5	10 (62.5)	3 (50.0)	2 (100)	1 (50.0)	4 (66.7)
Oddor Mean Chey	2	16	7 (43.8)	3 (50.0)	0	1 (50.0)	3 (50.0)	1.28	0	0	0	0	0
Koh Kong	2	16	0	0	0	0	0	0	3 (18.8)	2 (33.3)	0	0	1 (16.7)
Paillin	2	16	5 (31.3)	3 (50.0)	1 (50.0)	1 (50.0)	0	4.4	4 (25.0)	2 (33.3)	0	0	2 (33.3)
Bantheay Mean Chey	2	16	2 (12.5)	0	1 (50.0)	1 (50.0)	0	0.29	4 (25.0)	2 (33.3)	1 (50.0)	0	1 (16.7)
Pursat	2	16	5 (31.3)	1 (16.7)	2 (100)	1 (50.0)	1 (16.7)	8.6	2 (12.5)	2 (33.3)	0	0	0
Prey Veng	2	16	6 (37.5)	1 (16.7)	0	1 (50.0)	4 (66.7)	1.3	4 (25.0)	4 (66.7)	0	0	0
Svay Rieng	2	16	3 (18.8)	1 (16.7)	1 (50.0)	0	1 (16.7)	15.0	9 (56.3)	3 (50.0)		1 (50.0)	4 (66.7)
Mundulkiri	2	16	13 (81.3)	5 (83.3)	2 (100)	2 (100)	4 (66.7)	2.6	6 (37.5)	2 (33.3)	0	1 (50.0)	3 (50.0)
Ratanakiri	2	16	7 (43.8)	4 (66.7)	0	0	3 (50.0)	2.0	5 (31.3)	2 (33.3)	0	0	3 (50.0)
Steung Treng	2	16	4 (25.0)	1 (16.7)	0	0	3 (50.0)	10.1	8 (50.0)	3 (50.0)	1 (50.0)	1 (50.0)	3 (50.0)
Kratie	2	16	6 (37.5)	2 (33.3)	0	1 (50.0)	3 (50.0)	5.2	8 (50.0)	3 (50.0)	1 (50.0)	1 (50.0)	3 (50.0)
Kampong Thom	2	16	8 (50.0)	3 (50.0)	2 (100)	0	3 (50.0)	106.1	0	0	0	0	0
Preah Vihear	2	16	11 (68.8)	5 (83.3)	2 (100)	1 (50.0)	3 (50.0)	76.6	3 (18.8)	1 (16.7)	1 (50.0)	0	1 (16.7)
Total ^3^	52	496	214 (43.1)	84 (45.2)	26 (41.9)	19 (30.6)	85 (45.7)	23.2	155 (31.3)	78 (41.9)	12 (19.4)	7 (11.3)	58 (31.2)

^1^ Three markets were included in Phnom Penh (PP) and Siem Reap (SR), regarded as having the highest population, while two were included in the other 23 provinces. ^2^ The total number of each specimen was different in Phnom Penh and Siem Reap (18 chicken, 6 chicken cutting boards, 18 pork, and 6 pork cutting boards); Battambang (BB) and Preah Sihanouk (PSH) (12 chicken, 4 chicken cutting boards, 12 pork, 4 pork cutting boards), compared to other provinces (6 chicken, 2 chicken cutting boards, 6 pork, 2 pork cutting boards). ^3^ The total 496 samples included the 80 repeated samples of the 4 provinces/municipalities (PP, SR, BB, PSH) and excluded 36 samples from supermarkets. ^4^ Samples with MPN/g < 0.3, negative with *Salmonella* spp. were counted as 0, and not included in the average. MPN/g >110 was assigned randomly between 111 and 250 MPN/g for the calculation.

**Table 2:** The prevalence of *Salmonella* spp. and *Staphylococcus aureus* in chicken, pork, pork cutting boards and chicken cutting boards from traditional markets, supermarkets, in Cambodia and variation within one year.

Market Types	Total Positive Sample	Chicken (No. of Positive (%))	Chicken Cutting Board (No. of Positive (%))	Pork (No. of Positive (%))	Pork Cutting Board (No. of Positive (%))	*p-*Value ^4^
**Traditional Market**						
**Dry season ^1^ (n = 416)**		n = 156	n = 52	n = 156	n = 52	
*Salmonella* spp. *& S. aureus*	68	32 (20.5)	5 (9.6)	30 (19.2)	1 (1.9)	0.006
*Salmonella* spp.	169	63 (40.4)	22 (42.3)	70 (44.9)	14 (26.9)	0.150
*S. aureus*	144	72 (46.2)	11(21.2)	54 (34.6)	7 (13.5)	<0.001
**Wet season ^2^ (n = 80)**		n = 30	n = 10	n = 30	n = 10	
*Salmonella* spp. *& S. aureus*	9	6 (20.0)	0	3 (10.0)	0	-
*Salmonella* spp.	45	21 (70.0)	4 (40.0)	15 (50.0)	5 (50.0)	-
*S. aureus*	10	6 (20.0)	0	4 (13.3)	0	-
**Supermarkets ^3^ (n = 36)**		n = 18	-	n = 18		
*Salmonella* spp. *& S. aureus*	1	0	-	1 (5.6)	-	-
*Salmonella* spp.	10	3 (16.7)	-	7 (38.9)	-	-
*S. aureus*	1	0	-	1 (5.6)	-	-
**Overall (n = 532)**		n = 204	n = 62	n = 204	n = 62	
*Salmonella* spp. *& S. aureus*	78	38 (18.6)	5 (8.1)	34 (16.7)	1 (1.6)	0.166
*Salmonella* spp.	224	87 (42.6)	26 (41.9)	92 (45.1)	19 (30.6)	0.249
*S. aureus*	155/532	78 (38.2)	11 (17.7)	59 (28.9)	7 (11.3)	<0.001

^1^ The samples were from 2 markets in each of 23 provinces and 3 markets in Phnom Penh and Siem Reap. ^2^ The 80 repeated samples in the wet season were only from 4 provinces/municipalities, including Phnom Penh, Siem Reap, Battambong and Preah Shihanouk. ^3^ The samples were from 4 supermarkets in Phnom Penh and 2 supermarkets in Siem Reap and collected only in the dry season. ^4^ Chi-square test.

**Table 3:** Factors associated with prevalence of *Salmonella* spp. and *S. aureus* contamination and co-contamination in samples from Cambodian markets using logistic regression.

Pathogens	Variables	Odds Ratio	95% CI	Coefficient	S.E.	*p-*Value
*Salmonella* spp. *& Staphylococcus aureus*	Species (chicken compared to pork)	1.28	0.78–2.1	0.25	0.25	0.32
	Sample (meat compared to cutting board)	4.66	1.97–11.03	1.54	0.44	<0.001
	Market type (supermarket compared to traditional market)	0.11	0.01–0.84	−2.18	1.02	0.034
	Season (dry compared to wet season)	0.64	0.3–1.36	−0.45	0.38	0.24
	Constant			−3.05	0.44	<0.001
*Salmonella* spp.	Species (chicken compared to pork)	1.03	0.72–1.46	0.03	0.18	0.86
	Sample (meat compared to cutting board)	1.47	0.96–2.24	0.38	0.22	0.07
	Market type (supermarket compared to traditional market)	0.51	0.24–1.1	−0.67	0.39	0.09
	Season (wet compared to dry season)	1.89	1.16–3.06	0.63	0.25	0.01
	Constant			−0.69	0.21	0.001
*Staphylococcus aureus*	Species (chicken compared to pork)	1.60	1.07–2.37	0.47	0.2	0.021
	Sample (meat compared to cutting board)	3.55	2.05–6.15	1.27	0.28	<0.001
	Market type (supermarket compared to traditional market)	0.04	0.01–0.3	−3.2	1.02	0.002
	Season (wet compared to dry season)	0.26	0.12–0.51	−1.37	0.36	<0.001
	Constant			−1.89	0.28	<0.001

**Table 4:** Variables associated with Log CFU/g of coagulase-positive staphylococci in samples collected from Cambodian markets.

Variable	Coefficient	95% Confidence Interval	Std Error	*p-*Value
Market type (supermarket compared to traditional market)	−1.054	−1.471–−0.638	0.212	<0.001
Meat type (chicken compared to pork)	0.250	0.044–0.456	0.105	0.017
Sample type (meat compared to cutting board)	0.648	0.402–0.894	0.125	<0.001
Season (dry compared to wet)	−0.590	−0.880–−0.300	0.147	<0.001
Constant	0.927	0.516–1.338	0.209	<0.001

**Table 5:** Number of samples collected from traditional markets and supermarkets in Cambodia.

Sampling Round	Chicken Meat	Chicken Cutting Board	Pork Cutting Board	Pork
Traditional market, dry season ^1^	156	52	52	156
Traditional market, wet season ^2^	30	10	10	30
Supermarkets ^3^	18	-	-	18
Total specimen	204	62	62	204
			Total specimen = 532	

^1^ Three markets were included in Phnom Penh and Siem Reap, while two markets were included in the other 23 provinces.^2^ The total 80 samples were re-sampled from Battambang, Phnom Penh, Siem Reap, and Preah Sihanouk. ^3^ Four supermarkets in Phnom Penh and two supermarkets in Siem Reap.

## Data Availability

Not applicable.
